# The Neuroeconomics of Tobacco Demand: An Initial Investigation of the Neural Correlates of Cigarette Cost-Benefit Decision Making in Male Smokers

**DOI:** 10.1038/srep41930

**Published:** 2017-02-03

**Authors:** Joshua C. Gray, Michael T. Amlung, Max Owens, John Acker, Courtney L. Brown, Gene H. Brody, Lawrence H. Sweet, James MacKillop

**Affiliations:** 1 Department of Psychology, University of Georgia, Athens, GA 30602, USA; 2 Peter Boris Centre for Addictions Research, McMaster University/St. Joseph’s Healthcare Hamilton, 100 West 5th At., Hamilton, ON L8N 3K7, Canada; 3 Center for Integrated Healthcare, Syracuse VA Medical Center, Syracuse, NY 13210, USA; 4 Department of Health Psychology, University of Missouri, Columbia, MO 65211, USA; 5 Center for Family Research, University of Georgia, Athens, GA 30605, USA

## Abstract

How the brain processes cigarette cost-benefit decision making remains largely unknown. Using functional magnetic resonance imaging (fMRI), this study investigated the neural correlates of decisions for cigarettes (0–10 cigarettes) at varying levels of price during a Cigarette Purchase Task (CPT) in male regular smokers (*N* = 35). Differential neural activity was examined between choices classified as inelastic, elastic, and suppressed demand, operationalized as consumption unaffected by cost, partially suppressed by cost, and entirely suppressed by cost, respectively. Decisions reflecting elastic demand, putatively the most effortful decisions, elicited greater activation in regions associated with inhibition and planning (e.g., middle frontal gyrus and inferior frontal gyrus), craving and interoceptive processing (anterior insula), and conflict monitoring (e.g., anterior cingulate cortex). Exploratory examination in a harmonized dataset of both cigarette and alcohol demand (*N* = 59) suggested common neural activation patterns across commodities, particularly in the anterior insula, caudate, anterior cingulate, medial frontal gyrus, and dorsolateral prefrontal cortex. Collectively, these findings provide initial validation of a CPT fMRI paradigm; reveal the interplay of brain regions associated with executive functioning, incentive salience, and interoceptive processing in cigarette decision making; and add to the literature implicating the insula as a key brain region in addiction.

Cigarette smoking remains an onerous public health problem^1^ and is increasingly studied using behavioral economics, an integration of psychological and microeconomic approaches^2^. One fundamental behavioral economic index of decision making for addictive substances is how much of it an individual will consume at a given price (i.e., drug demand). Demand for a substance can be quantified using operant schedules that measure consumption at varying levels of behavioral effort^3^ or purchase tasks that measure estimated consumption at varying levels of price^4^. Higher cigarette demand on Cigarette Purchase Tasks (CPT)^5^ is associated with higher levels of daily smoking^6^,^7^, more severe nicotine dependence^6^,^8^, lower motivation to quit smoking^8^, and smoking over time^9^. In laboratory studies, state assessments of cigarette demand complement traditional self-report measures of craving in the assessment of acute motivation^10^,^11^,^12^.

The most comprehensive strategy for characterizing demand is demand curve analysis, which examines estimated consumption of a commodity across escalating prices^13^. An individual’s demand typically conforms to three behavioral patterns across the curve: (1) maximum consumption, during which there is insensitivity to price increases (i.e., inelastic demand); (2) decreasing consumption as price increases (i.e., elastic demand); (3) zero consumption, where costs have entirely outweighed benefits (i.e., suppressed demand). By providing a systematic characterization of the relationship between consumption and costs, drug demand is also a promising behavioral economic model for neuroeconomic analysis (i.e., the neuroscience of decision making). However, despite advances in other domains of decision making, only a small number of studies have examined the neural correlates of purchasing decisions for generic non-addictive goods^14^,^15^,^16^ and even fewer have explored purchasing decisions for addictive goods with systematic variation of price to examine different phases of the demand curve.

To date, two studies have directly investigated drug-related demand decision making. MacKillop *et al*. developed a functional magnetic resonance imaging (fMRI) purchase task paradigm to examine the neural correlates of alcohol demand in a sample of 24 heavy drinkers^17^. Two primary profiles of activation were observed for different aspects of decision making. The first included brain regions that were differentially active when making the decision to consume any alcohol relative to choosing not to consume alcohol. This profile was characterized by heightened activation in areas associated with attention (posterior parietal cortex), deliberation and decision making (medial prefrontal cortex, dorsolateral prefrontal cortex; dlPFC), introspection (posterior cingulate cortex), and drug cravings (anterior insula). The second profile represented activity specific to partially suppressed consumption (during the elastic period of the demand curve) compared to maximum consumption and no consumption, which was characterized by greater activation in areas associated with drug cravings (anterior insula), motivational drive (striatum), mathematical calculations (angular gyrus), and cognitive deliberation (dlPFC).

The second study employed a purchase task for cannabis ‘puffs’ in non-treatment seeking daily cannabis smokers^18^. Neural activity was divided between choices to purchase puffs and choices to decline puffs, revealing several overlapping regions with the previous alcohol demand study, including dorsal striatum, insula, posterior parietal cortex, anterior and posterior cingulate, and dorsolateral prefrontal cortex, all more active in decisions to purchase puffs.

The goal of the current investigation was to extend this line of inquiry to the neural correlates of tobacco demand. This experiment used an fMRI CPT paradigm in a sample of daily smokers to characterize differential neural activation during the three canonical periods of the demand curve (i.e., inelastic demand - choices unaffected by cost; elastic demand - choices partially affected by cost; and suppressed demand - choices completely affected by cost, meaning no cigarette consumption). In other words, the study sought to clarify differential brain activity during maximum preferred cigarette consumption, reduced cigarette consumption as a result of greater associated costs, and completely abolished consumption. Additionally, as an exploratory aim, the study utilized a conjunction mask approach previously applied to the neural correlates of intertemporal choice^19^ to compare the neural activation associated with cigarette demand to that of alcohol demand in the previous study^17^. This exploratory aim sought to refine the characterization of the common neural profiles underlying demand decision making and address the extent to which the patterns of activation are commodity-specific.

## Methods

### Participants

The inclusion criteria for participation in this study were: (1) male; (2) right-handed; (3) 18–55 years old; (4) self-reported smoking of >5 cigarettes per day; (5) baseline expired carbon monoxide (CO) >5 parts per million; (6) at least 10^th^ grade education; and (7) computer use >4 days per week. Only male participants were enrolled to avoid potential sex differences for which the study was not powered to systematically examine. Given the single sex design, males were selected because the prevalence of smoking is higher among men^20^. Exclusionary criteria were: (1) any head injury more severe than a mild traumatic brain injury (TBI) or >2 mild TBIs; (2) MRI contraindications; (3) receiving mental health services within the last six months or prescribed psychotropic medications; (4) actively seeking or having undergone smoking cessation treatment in past 90-days; (5) weekly illicit drug use, other than marijuana; and (6) living with someone who has participated in the study.

Participants were 35 non-treatment-seeking male daily smokers of European (80.0%), African (17.1%), and Asian (2.9%) ancestry. Mean age was 25.8 (SD = 6.4) and median income was $30,000 (interquartile range =< $15,000–$44,999). Participants reported smoking slightly less than a pack of cigarettes per day (*M* = 15.8, SD = 6.7) and were moderately nicotine dependent (Fagerström Test for Nicotine Dependence [FTND]^21^ *M* = 3.1, SD = 1.8). Mean expired carbon monoxide (CO) at the MRI scanning session was 18.2 (SD = 11.2) (Bedfont Scientific piCO + Smokerlyzer, Kent, UK). In terms of drug use, median use of marijuana in the past three months was less than monthly and other substance use was minimal. Nine additional participants were enrolled but were excluded from analysis for low effort (i.e., excessive missing data or inconsistent responding; *n* = 3), insufficient decision making variability (i.e., always selecting zero cigarettes, no intermediate consumption, *n* = 5), and failure to comply with the protocol (*n* = 1).

### Experimental Protocol

After a positive telephone screen interview, participants were invited to attend a one-hour in-person screening session to further assess eligibility and to obtain informed consent for the full study. Participants who met the criteria for the full study were instructed not to use illicit drugs or alcohol within 24 hours of their scheduled appointment, although this was not biochemically verified. At the start of the scan session, participants were asked to first smoke a cigarette to ensure a standard period of abstinence prior to the scan. The protocol prior to the scan lasted approximately two and a half hours and was comprised of an expired CO assessment (15–20 minutes after smoking the pre-session cigarette), questionnaires, and orientation to scanner paradigms. Participants then underwent a 60-minute MRI scan. At the end of the study, participants randomly selected a poker chip out of a fishbowl, each corresponding to one of the 88 questions on the CPT. Participants received the cigarettes (*M* = 3.80; SD = 3.76; maximum of 10 cigarettes), money (*M = *$9.35; SD = 0.66; maximum of $10), or both that were associated with the choice they made for this particular question. Participants were compensated $15 for the in-person screening and $40 for the fMRI session. The study was approved by the University of Georgia Institutional Review Board, all participants provided written informed consent, and the experiment was performed in accordance with guidelines and regulations of the IRB.

### fMRI Cigarette Purchase Task Paradigm

The CPT was administered in the MRI scanner as an event-related design using similar procedures to the previous alcohol demand study^17^. Four CPT runs were administered, with each run comprised of 22 trials lasting a total of 7 minutes per run (88 total trials). Subjects were provided with a $10 cigarette “tab” in cash, and were asked how many cigarettes (of their preferred brand) they would purchase at 22 different prices per cigarette ($0.00, $0.01, $ 0.02, $0.03, $0.04, $0.05, $0.09, $0.10, $0.14, $0.15, $0.19, $0.20, $0.21, $0.22, $0.23, $0.24, $0.25, $0.26, $0.28, $0.29, $0.30, $0.34, $0.35, $0.40, $0.50, $1.00, $2.00, $2.50, $5.00, $10.00). The distribution of prices was adapted from a former investigation^22^. The corresponding pack price (20 cigarettes per pack) was also provided on each item. The CPT stimuli, depicted in supplementary materials, were programmed in E-Prime 2.0 software^23^ and were viewed using an MRI-compatible stimulus presentation goggle system. Each trial was divided into two epochs: a *“Consider”* epoch (duration = 6 s) in which participants were instructed to contemplate their choice but were not able to respond, and a “*Choose*” epoch (duration ≤ 7 s) during which participants manually entered their choice using MRI-compatible response boxes. The division of the decision-making process into two epochs enables isolation of consideration from actual response execution (based on the two-stage model of decision making^24^) and is consistent with our prior work utilizing an alcohol purchase task^17^. During the *Choose* epoch, participants moved a green box to their desired number of cigarettes with their right hand and submitted their response with their left hand. After participants selected their response, the display changed to an active baseline inter-stimulus interval (ISI) screen (*M* duration = 5.5 s; range = 1–10 s) in which all numbers were replaced by Xs.

Events were classified into three mutually exclusive categories based on the canonical form of the demand curve. Specifically, choices were classified according to the previously used classification of “Inelastic”, “Elastic”, and “Suppressed”^17^. Inelastic choices were defined as each participant’s maximum selected number of cigarettes, which typically occurred at the lowest ranges of price per cigarette (e.g., free, 1¢). Elastic choices were defined as decisions in which participants selected fewer than their personal maximum, but still selected one or more cigarettes. Finally, Suppressed choices were defined as decisions for no cigarettes, reflecting that the price has outweighed the reinforcing value of cigarettes. This categorization putatively reflects systematic differences in decision-making cost-benefit profiles, with Inelastic choices reflecting the perception of benefits of smoking but not associated cost; Elastic choices reflecting perception of concurrent benefit and cost of smoking; and Suppressed choices reflecting the perception of cost of smoking but not benefit.

### Structural and Functional MRI Data Acquisition and Analysis

Structural imaging consisted of a high-resolution T_1_-weighted, fast spoiled gradient echo scan (repetition time [TR] = 7.8 ms, echo time [TE] = 3.1 ms, flip angle = 20°; field of view [FOV] = 25.6 cm, matrix = 256 × 256, voxel size = 1 mm^3^, 160 contiguous 1 mm axial slices). Functional imaging consisted of T_2_*-weighted echo planar imaging scans with a gradient echo pulse sequence (TR = 2000 ms, TE = 25 ms, flip angle = 90°, FOV = 22.5 cm, matrix = 64 × 64, voxel size = 3.5 mm^3^, 40 contiguous 3.5 mm axial slices). Three dummy samples preceded each functional scan to establish equilibrium.

Magnetic resonance imaging data processing and analysis were conducted using Analysis of Functional Neuroimages software (AFNI^25^) with follow-up analyses using SPSS 17.0 (IBM, Armonk, NY). Preprocessing steps included correction for slice timing acquisition, volume registration to the third volume of the first CPT run (i.e., to avoid initial movement due to startle from scanner startup), alignment with the anatomical dataset, transformation into Talairach space^26^, spatial smoothing using a 3.5 mm full-width half-maximum Gaussian filter, and scaling to percent signal change from mean signal intensity for each run. Individual runs were concatenated into a single dataset. Analysis of motion in the scanner verified that no participants moved excessively (>3.5 mm in any direction; *M* translation = 0.57 mm; *SD* = 0.53 mm).

Analysis of individual brain responses were performed using a general linear model (GLM) that consisted of six task-related regressors (Inelastic/Elastic/Suppressed for *Consider* and *Choose* epochs), seven nuisance regressors to account for missing trials (~2% of trials) and observed head motion (x, y, z, roll, pitch, yaw), and three regressors accounting for linear, quadratic, and cubic trends. BOLD signal was estimated using the canonical hemodynamic response function for each choice type for each of the two trial epochs. Of note, the ‘dmblock(1)’ function (from 3dDeconvolve in AFNI) was utilized to scale all events to the same relative amplitude of 1.

Group analyses were conducted using GLM *β* coefficients. Differential activity by trichotomous event type (Inelastic, Elastic, Suppressed) was calculated using an empirically-defined disjunction mask approach^17^,^27^. This approach uses voxel-wise t-tests to isolate relevant decision-making voxels that were significantly activated or deactivated relative to baseline in at least one of the three choice types. To minimize the likelihood of false positives, 1000 Monte Carlo simulations were run using Alphasim in AFNI, incorporating the average spatial smoothness in the data in the x, y, and z planes. A family-wise *α* < 0.05 required a threshold of *p* < 0.0001 and minimum cluster size of 5 voxels. Of note, these t-tests were conducted on a group mask comprised of voxels present in >70% of participants. The subsequent disjunction mask generated was then examined on a voxel-wise basis for clusters exhibiting significantly differential average BOLD signal by choice type using AFNI program 3dANOVA2, which included a three-level fixed factor for decision type and participant as a random factor. To control Type I error rate, a false discovery rate (FDR) of *q* = 0.01 was applied^28^. The mean *β* coefficient values were subsequently extracted from each cluster (>5 voxels) that survived error correction and paired samples t-tests were then used to examine contrasts between each choice type (i.e., Inelastic vs. Elastic, Elastic vs. Suppressed, and Inelastic vs. Suppressed).

For the exploratory aim of examining combined activation from the current study and the previous study using an fMRI Alcohol Purchase Task (APT)^17^, the conjunction analysis technique applied by McClure *et al*. was used^19^. Specifically, the data from the previous study were re-processed using identical specifications to the current study and two conjunction masks of voxels active by condition (i.e., *Consider* and *Choose*) in both studies was generated. The conjunction masks were created by generating a *Consider* mask, comprised of neural activation from both studies during this condition, and a *Choose* mask, comprised of neural activation from both studies during this condition. Both masks captured voxels active during any of the three phases of demand as compared to rest. Of note, the previous study was conducted at the same MRI facility with the same staff and a paradigm that was visually almost identical.

## Results

### Behavioral Results

**Figure 1:**
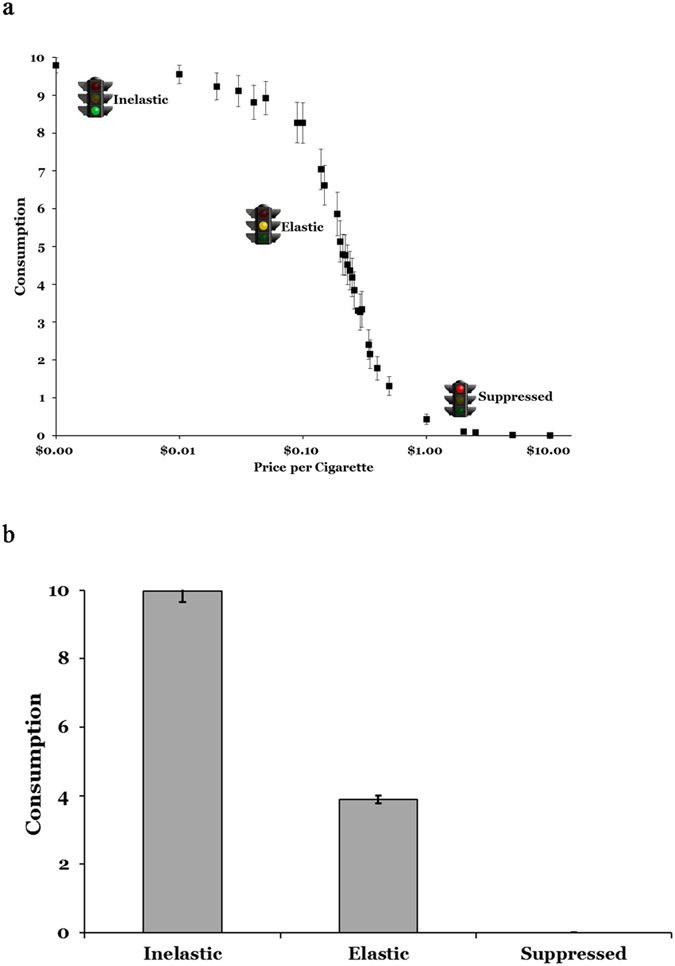
Behavioral performance during the fMRI cigarette purchase task. Choices were classified into three categories. “Inelastic” choices were defined as a participant’s maximum selected number of cigarettes. “Elastic” choices were trials in which a participant selected some number of cigarettes, but fewer than their personal maximum, reflecting competing valuation of money versus cigarettes. “Suppressed” choices were choices for no cigarettes, reflecting the cost outweighing the reinforcing value of the cigarettes. Panel a presents the empirical cigarette demand curve from participant performance on the CPT. Average consumption levels are denoted with squares with respective bars representing standard error of measurement (SEM). Panel b presents the average number of cigarettes (±SEM) associated with three choice types used in the analyses. Traffic lights were obtained from http://www.clker.com/.

Behavioral performance on the CPT conformed to a prototypic demand curve (Fig. 1a) and behavioral choices following the trichotomization revealed the anticipated substantial differences (Fig. 1b). The mean number of trials for each choice type was Inelastic, *M* = 26.17, SE = 2.40; Elastic *M* = 37.17; SE = 3.18; Suppressed, *M* = 23.20, SE = 2.96; and missing, *M* = 1.46; SE = 2.62. There were significant main effects of choice type on response latency (*F*(2, 68) = 147.49, *p* < 0.001, *η*^*2*^_*p*_ = 0.81), with participants responding fastest during Suppressed (*M* = 1139.17 ms; SE = 30.51), followed by Elastic (*M* = 1465.52 ms; SE = 8.97), and then Inelastic choices (*M* = 1584.32 ms; SE = 13.33; contrast *p*s < .001).

### Neural Correlates of Cigarette Demand during the *Consider* Epoch

**Figure 2:**
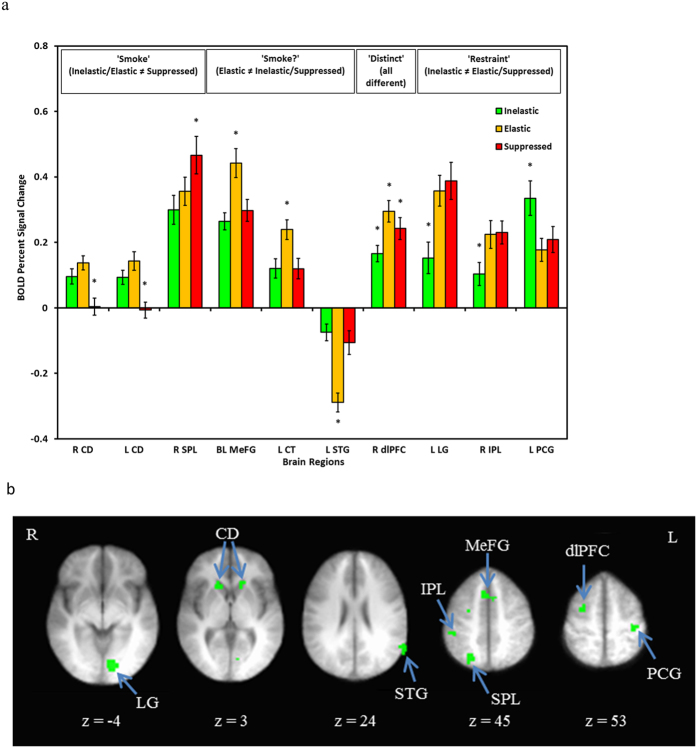
BOLD signal associated with differential activity during the *Consider* epoch of the CPT. Panel a presents individual regions organized by common patterns of activation (e.g., ‘Smoke’ reflects greater activation during Inelastic and Elastic demand compared to Suppressed demand). * = choice type is significantly different than the other two choice types. Panel b presents axial slices depicting the locations of the differentially active brain regions, with the exception of the cerebellar tonsil. Radiological conventions are used and side of brain is indicated by R or L (right, left). CD = caudate; SPL = superior parietal lobule; MeFG = medial frontal gyrus; CT = cerebellar tonsil; STG = superior temporal gyrus; dlPFC = dorsolateral prefrontal cortex; LG = lingual gyrus; IPL = inferior parietal lobule; PCG = postcentral gyrus.

Ten regions were identified which significantly discriminated by choice type in the *Consider* epoch (Fig. 2). Three of these regions conformed to a pattern described as ‘Smoke’ (i.e., significantly different activity for Inelastic and Elastic, involving maximum or some consumption, vs. Suppressed choices, involving no consumption). Specifically, bilateral caudate exhibited significantly greater activity for Smoke choices, whereas right superior parietal lobule indicated significantly greater activity for Suppressed choices. Three additional regions conformed to a pattern referred to as ‘Smoke’? (i.e., significantly different activity for Elastic choices compared to Inelastic or Suppressed, reflecting ambivalence about level of consumption). These included bilateral medial frontal gyrus (MeFG) and left cerebellar tonsil, all exhibiting significantly greater activation for Elastic choices. Additionally, left STG exhibited significantly greater deactivation for Elastic choices. Similar to the ‘Smoke’? pattern, the right dlPFC exhibited greatest activation during Elastic choices, followed by Suppressed, and then Inelastic (i.e., ‘Distinct’, a pattern of overall differentiation across choice types). Three regions were consistent with a pattern reflecting price effects on demand (i.e., differential activation in Elastic and Suppressed choices as compared to Inelastic), referred to as a ‘Restraint’ pattern. These included left lingual gyrus and right inferior parietal lobule (IPL) demonstrating significantly greater activation in Elastic and Suppressed choices, and left postcentral gyrus which had significantly greater activation during Inelastic choices. Full montages of these patterns are included in supplementary materials (Figure S4).

### Neural Correlates of Cigarette Demand during the *Choose* Epoch

**Figure 3:**
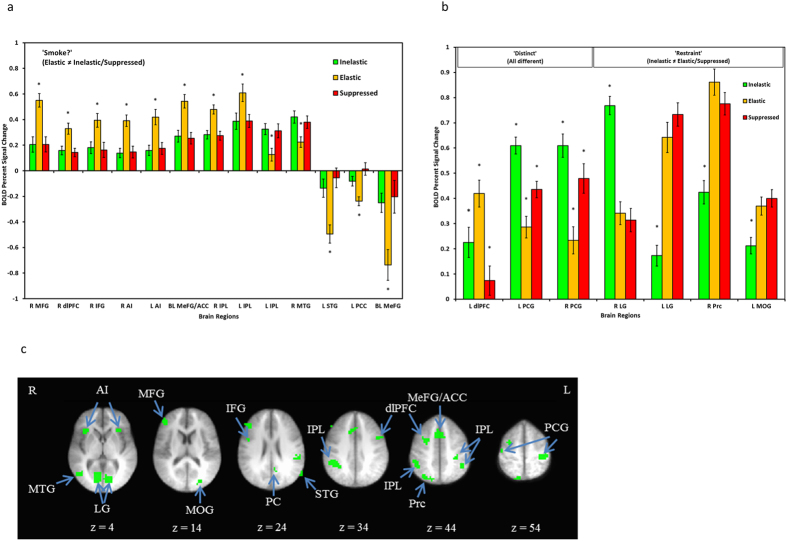
BOLD signal associated with differential activity during the *Choose* epoch of the CPT. Regions are organized by common patterns of differential activation. Panel a presents the regions exhibiting the ‘Smoke’? profile (i.e., different activity during Elastic choices compared to Inelastic or Suppressed choices). * = choice type is significantly different than the other two choice types. Panel b presents regions associated with the ‘Distinct’ profile (i.e., different levels of activity across all choice types) and ‘Restraint’ profile (i.e., differential levels of activity during Inelastic choices). Panel c presents axial slices depicting the locations of the differentially active brain regions. Radiological conventions are used and side of brain is indicated by R or L (right, left). MFG = middle frontal gyrus; dlPFC = dorsolateral prefrontal cortex; IFG = inferior frontal gyrus; AI = anterior insula; ACC = anterior cingulate cortex; IPL = inferior parietal lobule; MTG = middle temporal gyrus; STG = superior temporal gyrus; PCC = posterior cingulate cortex; PCG = postcentral gyrus; LG = lingual gyrus; Prc = precuneus; MOG = middle occipital gyrus.

Twenty regions significantly discriminated by choice type in the *Choose* epoch (Fig. 3). The dominant pattern across thirteen regions was activation conforming to the ‘Smoke’? pattern. Significantly greater activity was identified in Elastic choices in the right MFG, right dlPFC, right inferior frontal gyrus (IFG), bilateral insula, bilateral MeFG/anterior cingulate cortex (ACC), and bilateral parietal lobule, while significantly greater activation in Inelastic and Suppressed choices was evident in the left IPL and right middle temporal gyrus. Furthermore, significantly greater deactivation in Elastic choices was present in the left STG, left PCC, and right MeFG. Three regions fit the ‘Distinct’ pattern. Left dlPFC had significantly greater activation during Elastic choices, followed by Inelastic choices, and then Suppressed choices. Additionally, bilateral postcentral gyrus had significantly greater activity during Inelastic choices followed by Suppressed choices and then by Elastic choices. The final pattern of activation in four regions was consistent with ‘Restraint,’ including greater activation during Inelastic choices in the right lingual gyrus and greater activation during Elastic and Suppressed choices in the left lingual gyrus, right precuneus, and middle occipital gyrus. Full montages of these patterns are included in supplementary materials (Figure S5).

For exploratory purposes, the associations between elasticity, the demand parameter reflecting overall price sensitivity, and brain activity in empirically-defined regions that were selectively more active during Elastic decisions (i.e., the first 8 regions in Fig. 3a) was examined. The elasticity parameter was selected because it indexes an aggregate property of the demand curve, it reflects the most behaviorally conflicted phase of preferences, and it was least constrained by task-specific parameter. Elasticity was derived using Hursh & Silberberg’s non-linear exponential demand model^29^ and was base-10 log transformed to reduce skewness; two participants were excluded for poor model fit. Controlling for income, a significant relationship was present between elasticity and the left anterior insula (*r* = −0.427, *p* = 0.015). Finally, given the magnitude of the insula association and previous evidence for insular activation scaling with task difficulty^30^, insular activation was also examined with reaction time during Elastic trials, revealing a positive relationship between left anterior insula activation and Elastic decision reaction time (*r = *0.436, *p* = 0.008). Although elasticity and reaction time were not correlated with each other (*r* = −0.101, *p* = 0.576), when elasticity and reaction time were both entered into a regression model, only elasticity remained significant (elasticity: *r* = −0.427, *p* = 0.015; reaction time: *r* = −0.010, *p* = 0.957), suggesting that the variance was best accounted for by elasticity.

### Conjunction Analysis

**Figure 4:**
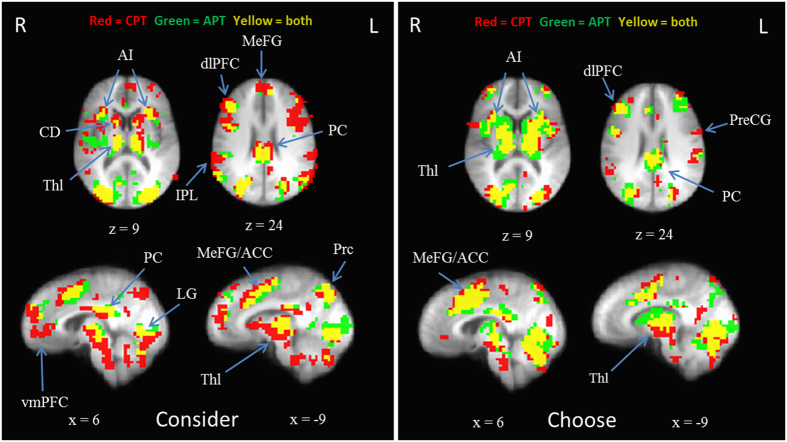
Mask of overlapping and discriminating voxels between the Cigarette Purchase Task (CPT) the Alcohol Purchase Task (APT) during the *Consider* (left) and *Choose* (right) epochs (*N* = 59; p < 0.0001, minimum cluster size = 5 voxels). Radiological conventions are used and side of brain is indicated by R or L (right, left). AI = anterior insula; CD = caudate; Thl = thalamus; dlPFC = dorsolateral prefrontal cortex; IPL = inferior parietal lobule; MeFG = medial frontal gyrus; ACC = anterior cingulate cortex; PC = posterior cingulate; vmPFC = ventral medial prefrontal cortex; Prc = precuneus; LG = lingual gyrus; PreCG = precentral gyrus.

The conjunction analysis suggested moderate convergence across *Consider* and *Choose* epochs from the previous alcohol study and the current tobacco study (Fig. 4). The regions implicated in cost-benefit drug decision making across both studies and present in both *Consider* and *Choose* epochs were the bilateral anterior insula, right dlPFC, bilateral PCC, MeFG/ACC, lingual gyrus, thalamus, and visual cortex. The regions that appeared to be discordant across studies were the ventral medial prefrontal cortex (vmPFC), IPL, and the precentral gyrus, all of which were only active in the present study. Exclusive overlap across the two studies is presented in supplementary materials (Figure S6). On the whole, regions that were present in the *Consider* epoch were also present in the *Choose* epoch, and vice versa, although bilateral vmPFC, caudate, and precuneus appear to be specifically recruited during the deliberative *Consider* epoch.

## Discussion

This study was the first to characterize the neural correlates of cigarette cost-benefit decision making. Broadly speaking, the brain regions found to be differentially active across choice types were reflective of incentive salience and valuation, inhibitory control, action selection, and visual processing. These regions parallel the underlying cognitive processes that putatively are engaged during this type of decision making: participants perceive the contingencies, register the values of the commodities involved, juxtapose benefits and costs of the options, and select a behavioral output (i.e., execute the decision). During the deliberative *Consider* condition, caudate activity was significantly greater when pursuing cigarettes (Inelastic and Elastic decisions), which is consistent with its role in goal-directed action and reward anticipation^31^. Greater activation of the superior parietal lobule during Suppressed choices is consistent with the finding that it stores cost-benefit signal differential^32^. Elastic choices (i.e., the ‘Smoke’? profile) were reflected in increased activation in MeFG^33^, potentially indicating increased conflict processing, and significantly more deactivation in the STG, suggesting greater disengagement of the default mode network^17^,^34^. Similarly, the dlPFC was most active in Elastic choices, followed by Suppressed, and then Inelastic, suggesting it is most recruited during Elastic choices, which involve both inhibitory processes^35^ and continued desire for cigarettes. Finally, in the ‘Restraint’ pattern, significantly greater activity during Elastic and Suppressed decisions in the lingual gyrus and IPL likely reflects greater visual attention under increased cognitive load^36^ and increased inhibition^35^, respectively.

During the *Choose* condition, in regions exhibiting a ‘Smoke’? profile, significantly greater activation in the MFG, IFG, and parietal lobule was present, suggesting deliberation, response inhibition and restraint^35^; MeFG/ACC suggesting conflict monitoring and action-outcome evaluation^37^,^38^,^39^; and anterior insula likely due to its involvement in interoceptive processing and craving^40^. Also of note, there was significantly greater deactivation of the default mode network (i.e., STG and PCC) during Elastic choices^34^,^41^. In contrast, the ‘Distinct’ pattern of activation for postcentral gyrus is likely to be reflective of simply greater sensorimotor activity required to select the minimum or maximum on the response screen. The primary regions of ‘Restraint’ patterns were related to visual processing, and broadly suggest greater visual attention during difficult Elastic and Suppressed decisions. Greatest precuneus engagement for Elastic followed by Suppressed decisions may be an indicator of self-referential processing, in which participants are making more effort to imagine oneself smoking at higher prices and considering its value^42^.

The second goal of the study was to integrate the current data with previous neuroimaging data on alcohol cost-benefit decision making to clarify the common neural correlates for this form of decision making across multiple drugs. These analyses focused on task general activation (i.e., neural activity present during any of the choice types) in each epoch (i.e., *Consider* and *Choose*) in an effort keep statistical tests manageable and to focus on the similarities and differences across substances (i.e., cigarettes and alcohol), rather than focus on differences across choice types or epochs. Both studies implicated regions involved in the processes discussed: inhibition, attention, reward anticipation, incentive salience, and action selection. For example, the PCC, within the dorsal subdivision, is likely an indication of its role in attentional focus, reward valuation, and its connectivity with frontal and parietal regions of cognitive control^43^,^44^,^45^,^46^. The regions encompassing dlPFC, MeFG, and ACC were an indication of attention, value encoding, and action-outcome evaluation^33^,^37^,^38^,^39^. Caudate activity during the *Consider* epoch is consistent with its role in reward anticipation^31^. The anterior insula was clearly recruited across both studies which is likely attributable to its role as an interoceptive mechanism that integrates self-state in order to influence reward valuation. In other words, it is involved in generating how much one “feels” like smoking or drinking at varying prices^40^.

Notably, all of these regions which were implicated in the conjunction mask were also recruited in the recent study which utilized a cannabis purchase task^18^, suggesting these common regions provide a core list of consistently relevant brain regions which occur during cost-benefit decision making involving drugs and money. The exception to this was the vmPFC, which was only present in the current study (during the *Consider* epoch) despite its prominent role in subjective valuation of stimuli^47^. This discrepancy is may be attributable to signal fallout^48^ and reduced power in the other two studies (see additional discussion in next paragraph). The IPL and the precentral gyrus were also clearly more recruited in the present study (as well as the cannabis study), which is suggestive of greater mathematical computation during the CPT and cannabis purchase task (perhaps a function of comparing prices to standard local pack prices and standard amounts spent per gram of cannabis). Of note, although these studies utilized similar paradigms, the levels of dependence were likely non-equivalent as the samples were heavy social drinkers, daily marijuana users, and daily cigarette smokers. Nonetheless these findings demonstrate similar and unique patterns of activation across a range of purchasing decisions for substances in very active drug users.

Despite the recruitment of the majority of brain regions implicated in goal-direction decision making and drug reward, two regions frequently recruited in reward-based decision making have been absent from this study, the alcohol study^17^, and the cannabis study^18^: the orbitofrontal cortex (OFC) and the ventral striatum. Given the recruitment of the vmPFC (typically recruited in parallel with the OFC for subjective valuation) in the present study, the absence of OFC activation is potentially due to signal loss^47^,^48^. The absence of activation identified in the ventral striatum is interesting given a recent meta-analysis finding it is robustly activated in subjective value computation^46^. This discrepancy may be attributable to the findings that as drug use becomes more habitual, the activity shifts from ventral to dorsal striatal activity, signifying a transition from ‘drug liking’ to ‘drug wanting’^49^.

The anterior insula was consistently recruited across the current and previous studies. This region has been previously identified as being central to homeostatic awareness, interoception, and integration of feeling^50^ and has also been found to play an important role in addiction and decision making^40^. Additionally, recent work found the anterior insula to consistently scale with difficulty in several perceptual decision-making tasks^30^. Taken together, this research indicates that in the context of addiction, anterior insula activation is likely a representation of how much one “feels” like pursuing a substance versus an alternative reinforcer. This is consistent with our follow-up analyses that found anterior insula activation was associated with higher overall cigarette demand. In other words, anterior insula activation is theorized to be particularly high (during Elastic choices) in individuals with high cigarette demand because they experience a greater degree of interoceptive conflict between their valuation of monetary reinforcers and their desire for cigarettes.

Three features of the behavioral data also merit further discussion. First, despite being a theoretically less difficulty decision, Inelastic response latency is marginally higher than Elastic response latency (consistent with the previous investigation^17^). This is likely due to the increased distance the cursor has to travel to get to the maximum. Second, as is the nature of event-related designs, there were significantly more Elastic trials. However, there was still adequate sampling of all of the choice types as evidenced by the substantial number of significant regions present in the contrasts even after several false discovery rate corrections. Third, participants generally started selecting less than the maximum number of cigarettes at around $0.10 per cigarette (see Fig. 1a) and generally spent less than their reported average price per pack (*M* = $4.77, SD = $0.91; or $0.24 per cigarette). In other words, participants exhibited an irrational choice strategy: they did not buy the maximum cigarettes even when the price was lower than market price. This may be for a number of reasons. Given the relatively rapid response times, it is unlikely that participants were engaging in deliberative mental math. Furthermore, the unit of choice (individual cigarettes) was more granular than typical pack-level decisions, which also may have skewed preferences. Therefore, the observed preferences may be because participants were making decisions based on heuristics or “gut feelings”, not purely rational choices to maximize the allocation of their resources.

These findings should be considered in the context of the study’s strengths and limitations. One clear strength was that the fMRI CPT paradigm had robust internal validity, mapping onto the standard behavioral paradigm and providing one actual outcome of money or cigarettes. In terms of limitations, however, the sample size was moderate and, as an initial proof-of-concept study, only daily smokers were enrolled, preventing valid between-subjects analyses. Because of power considerations, all participants were male, which makes generalizability to females uncertain. Furthermore, the economic constraints on the fMRI paradigm made it so participants could not purchase more than 10 cigarettes, spend more than $10 on cigarettes, and at costs of >$1 per cigarette, could not purchase the maximum number of cigarettes (e.g., only 1 cigarette could be purchased at $10 per cigarette). Therefore participants were unable to be inelastic at price points higher than $1 per cigarette. Although this was not a major threat in this study because participant choices typically became elastic much sooner than $1 (see Fig. 1a), a higher monetary tab can avoid this possible limitation altogether in future studies. Additionally, although the division between *Consider* and *Choose* conditions is theoretically supported by a two-stage model of decision making^24^, there is likely overlap across these two phases. The average within-subject voxel-wise correlation across the *Consider* and *Choose* phases by choice type (Inelastic *r* = 0.311; Elastic *r* = 0.424; Suppressed *r* = 0.297) suggests that these are related but distinct stages of decision making. Finally, some of the analyses were exploratory and therefore should be interpreted with caution. For example, the correlation between the insula and elasticity was based on an empirically-defined ROI, representing an extension of the task-related patterns of neural activity, not an independent finding. Similarly, the conjunction analyses were necessarily qualitative and thus definitive conclusions cannot be drawn about the overlapping neural activation across studies.

Acknowledging these limitations, the current study nonetheless provides initial support for characterizing cigarette cost-benefit decision making using fMRI. Further validation of this paradigm will be important, but these findings serve as a critical foundation for future studies investigating the neural correlates of differences between groups (e.g., nicotine dependent individuals vs. tobacco chippers) or following experimental manipulations that affect the value of cigarettes in clinical populations. This study contributes to the small but growing body of work using a neuroeconomic framework to understand the neural underpinnings of maladaptive decision making in nicotine dependence.

## Additional Information

**How to cite this article**: Gray, J. C. *et al*. The Neuroeconomics of Tobacco Demand: An Initial Investigation of the Neural Correlates of Cigarette Cost-Benefit Decision Making in Male Smokers. *Sci. Rep.* **7**, 41930; doi: 10.1038/srep41930 (2017).

**Publisher's note:** Springer Nature remains neutral with regard to jurisdictional claims in published maps and institutional affiliations.

## Supplementary Material

Supplementary Materials
