# Supplementary material for: The Neuroeconomics of Tobacco Demand: An Initial Investigation of the Neural Correlates of Cigarette Cost-Benefit Decision Making in Male Smokers
Source: Sci Rep. 2017 Feb 3;7:41930. doi: 10.1038/srep41930 (PMC5291221; doi:10.1038/srep41930)
Supplement: Supplementary Materials [file srep41930-s1.pdf]

**The Neuroeconomics of Tobacco Demand:**

**An Initial Investigation of the Neural Correlates of Cigarette Cost-Benefit Decision Making in Male Smokers**

Joshua C. Gray, MS; Michael T. Amlung, PhD; Max Owens, MS; John Acker, MS; Courtney L. Brown, PhD; Gene Brody, PhD; Lawrence H. Sweet, PhD; & James MacKillop, PhD

Supplementary Materials

1. **Supplementary Figures**
    a. Supplementary Figure 1. Schematic of the Cigarette Purchase Task (CPT) paradigm.
    b. Supplementary Figure 2. Voxel-wise contrasts of each choice condition (Inelastic, Elastic, Suppressed) to visually-matched rest during the *Consider* epoch.
    c. Supplementary Figure 3. Voxel-wise contrasts of each choice condition (Inelastic, Elastic, Suppressed) to visually-matched rest during the *Choose* epoch.
    d. Supplementary Figure 4. Montage of *Consider* contrasts for ‘Smoke’, ‘Smoke?’ and ‘Restraint’ patterns.
    e. Supplementary Figure 5. Montage of *Choose* contrasts for ‘Smoke?’ and ‘Restraint’ patterns.
    f. Supplementary Figure 6. Mask of overlapping voxels between the current study paradigm (i.e., the Cigarette Purchase Task [CPT]) and the previous study paradigm (i.e., the Alcohol Purchase Task [APT]) during the *Consider* (left) and *Choose* (right) epochs. ($p$ <.0001, minimum cluster size = 5 voxels).
2. **Supplementary Tables**
    a. Supplementary Table 1. Cigarette Purchase Task fMRI paradigm price sequences.
    b. Supplementary Table 2. *Consider* epoch ROIs revealing differential activation by choice type.
    c. Supplementary Table 3. *Choose* epoch ROIs revealing differential activation by choice type.

## 1. Supplementary Figures

**Supplementary Figure 1. Schematic of the Cigarette Purchase Task (CPT) paradigm.** Participants selected how many cigarettes they would consume (0-10) at varying levels of price ($0-$10/cigarette). During each decision, paradigm used two epochs, a fixed duration *Consider* epoch during which the participant was asked to consider their preference and a variable duration *Choose* epoch during which participants entered their preference. Following the response, the *Choose* epoch terminated and reverted to the rest stimulus, hence the variable duration. The paradigm used an active rest stimulus with all the visual properties of the active trials and jittered interstimulus interval (ISI) consisting of the rest stimulus for the residual time remaining in the *Choose* epoch plus an additional rest period of variable duration. RT = response time. The latter two panels in the schematic are identical and the combination of post-response (7s – RT) and ISI (1-10s) reflects the total ISI.

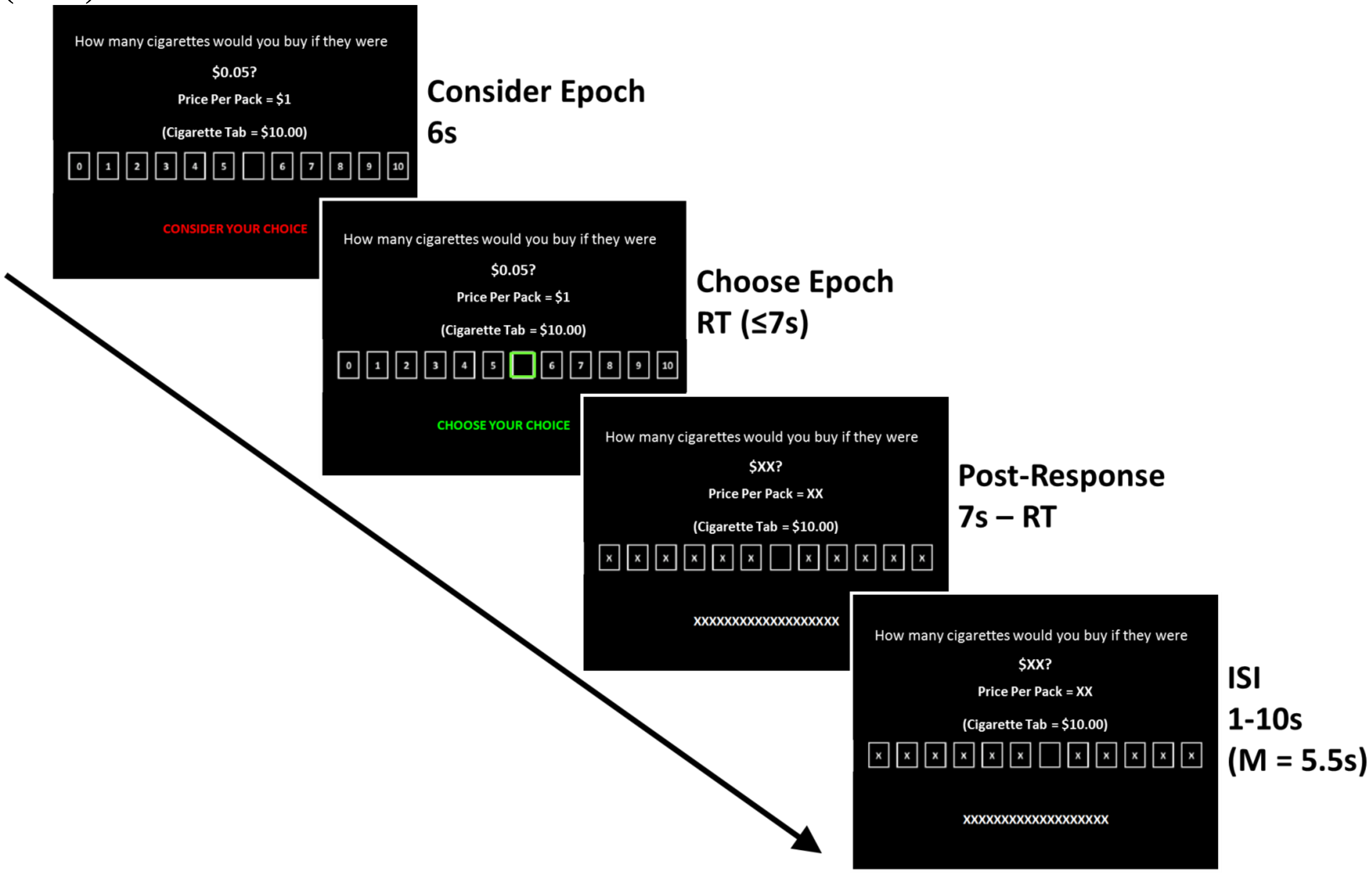

**Supplementary Figure 2. Voxel-wise contrasts of each choice condition (Inelastic, Elastic, Suppressed) to visually-matched rest stimuli during the *Consider* epoch.** Contrasts are thresholded at $p < .0001$ and cluster sizes of $\geq$ 5 voxels, reflecting an $\alpha < .05$. Panel A presents activation during Inelastic choices (i.e., the individual's maximum consumption choices). Panel B presents activation during Elastic choices (i.e., choices for less than maximum cigarettes, reflecting partial suppression of consumption by price). Panel C presents activation during Suppressed choices (i.e., zero consumption, reflecting price completely abolishing consumption).

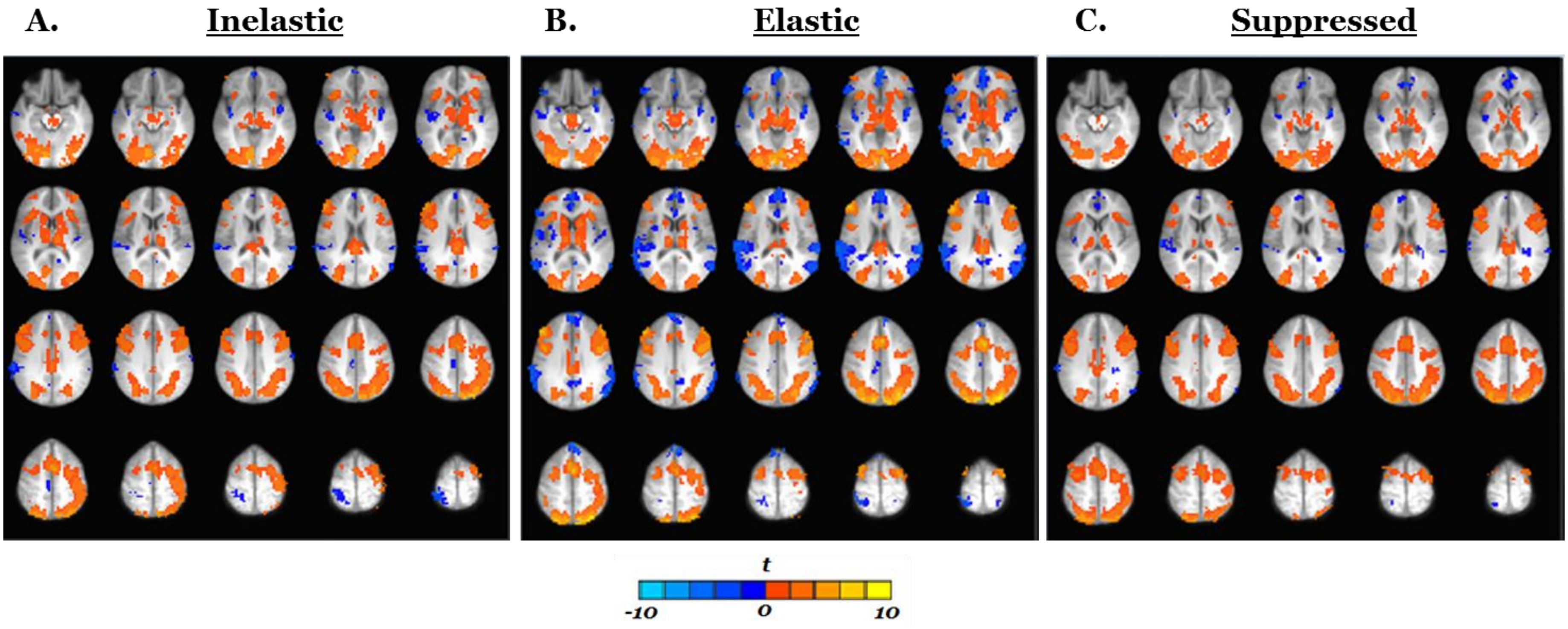

**Supplementary Figure 3. Voxel-wise contrasts of each choice condition (Inelastic, Elastic, Suppressed) to visually-matched rest during the *Choose* epoch.** Contrasts are thresholded at $p$ <.0001 and cluster sizes of $\geq$ 5 voxels, reflecting an $\alpha$ <.05. Panel A presents activation during Inelastic choices (i.e., the individual's maximum consumption choices). Panel B presents activation during Elastic choices (i.e., choices for less than maximum cigarettes, reflecting partial suppression of consumption by price). Panel C presents activation during Suppressed choices (i.e., zero consumption, reflecting price completely abolishing consumption).

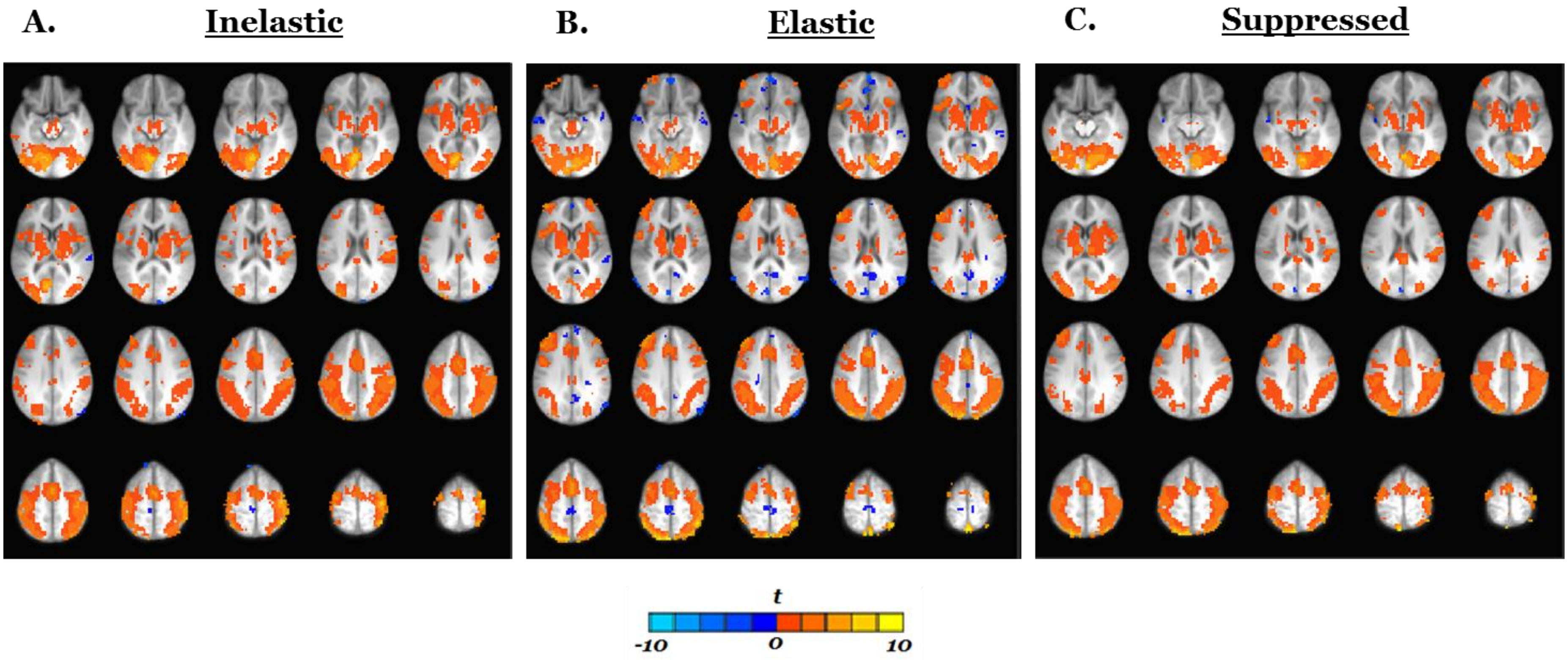

**Supplementary Figure 4. Montage of *Consider* contrasts for ‘Smoke’, ‘Smoke?’ and ‘Restraint’ patterns.** Panel A presents the ‘Smoke’ contrast (i.e., Inelastic/Elastic ≠ Suppressed). Orange indicates Suppressed choices elicited significantly greater activity. Blue indicates Suppressed choices elicited significantly less activity. Panel B presents the ‘Smoke?’ contrast (i.e., Elastic ≠ Inelastic/Suppressed). It also includes R MFG from ‘Distinct’ because the pattern of activation is comparable to the other ‘Smoke?’ regions. Orange indicates Elastic choices elicited significantly greater activity. Blue indicates Elastic choices elicited significantly greater deactivation. Panel C presents the ‘Restraint’ contrast (i.e., Inelastic ≠ Elastic/Suppressed). Orange indicates Inelastic choices elicited significantly greater activity. Blue indicates Inelastic choices elicited significantly less activity.

**A.** **‘Smoke’** **B.** **‘Smoke?’** **C.** **‘Restraint’**

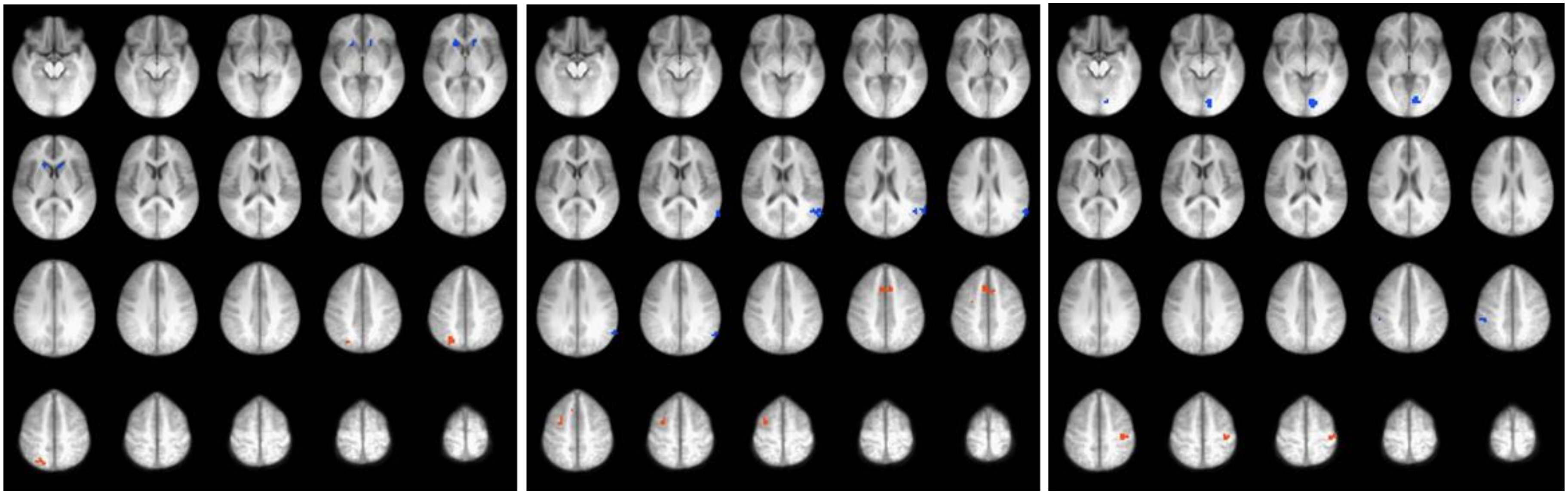

**Supplementary Figure 5. Montage of *Choose* contrasts for 'Smoke?' and 'Restraint' patterns.** Panel A presents the 'Smoke?' contrast (i.e., Elastic ≠ Inelastic/Suppressed). It also includes L MFG, L PCG, and R PCG from 'Distinct' because the pattern of activation is comparable to the other 'Smoke?' regions. Orange indicates Elastic choices elicited significantly greater activity. Blue indicates Elastic choices elicited significantly less activation or significantly greater deactivation. Panel B presents the 'Restraint' contrast (i.e., Inelastic ≠ Elastic/Suppressed). Orange indicates Inelastic choices elicited significantly greater activity. Blue indicates Inelastic choices elicited significantly less activity.

**A.** **'Smoke?'**

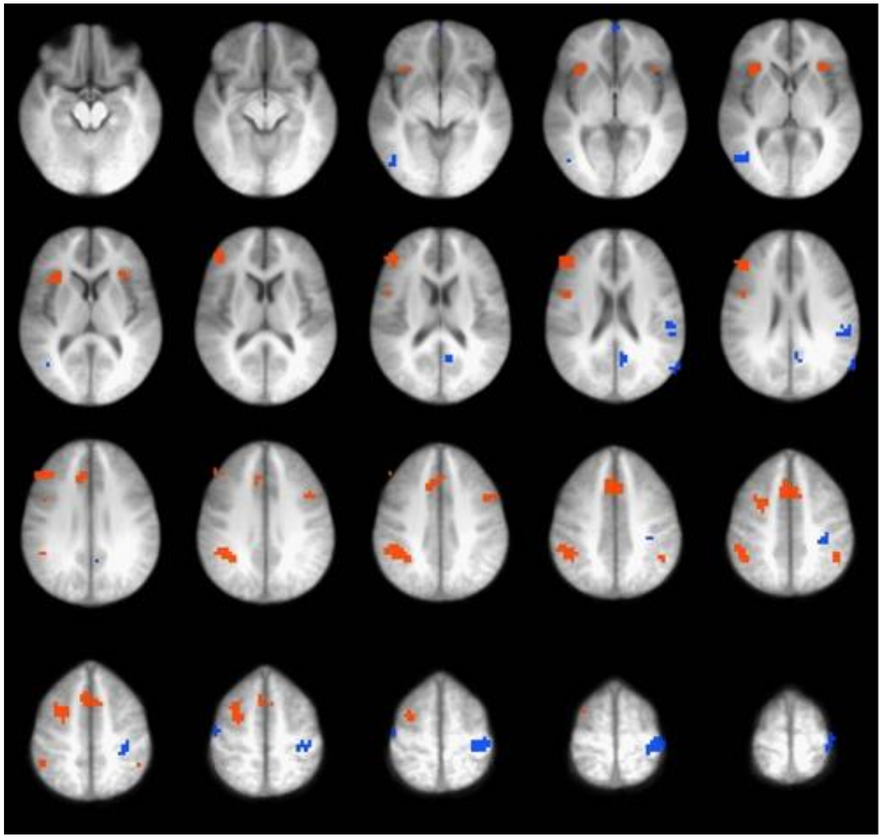

**B.** **'Restraint'**

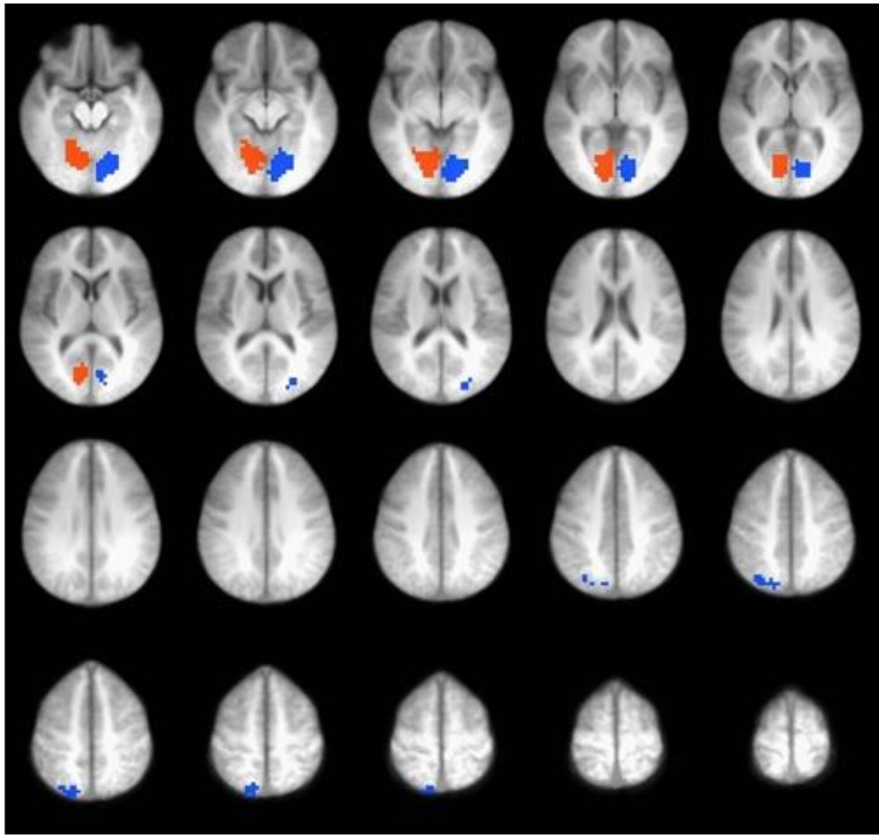

**Supplementary Figure 6. Conjunction masks of overlapping voxels between the Cigarette Purchase Task fMRI paradigm and the Alcohol Purchase Task fMRI paradigm during the *Consider* (left) and *Choose* (right) epochs ($N$ = 59; $p$ < .0001, minimum cluster size = 5 voxels)**. Radiological conventions are used and side of brain is indicated by R or L (right, left). AI = anterior insula; CD = caudate; Thl = thalamus; dlPFC = dorsolateral prefrontal cortex; MeFG = medial frontal gyrus; ACC = anterior cingulate cortex; PC = posterior cingulate; Prc = precuneus; LG = lingual gyrus.

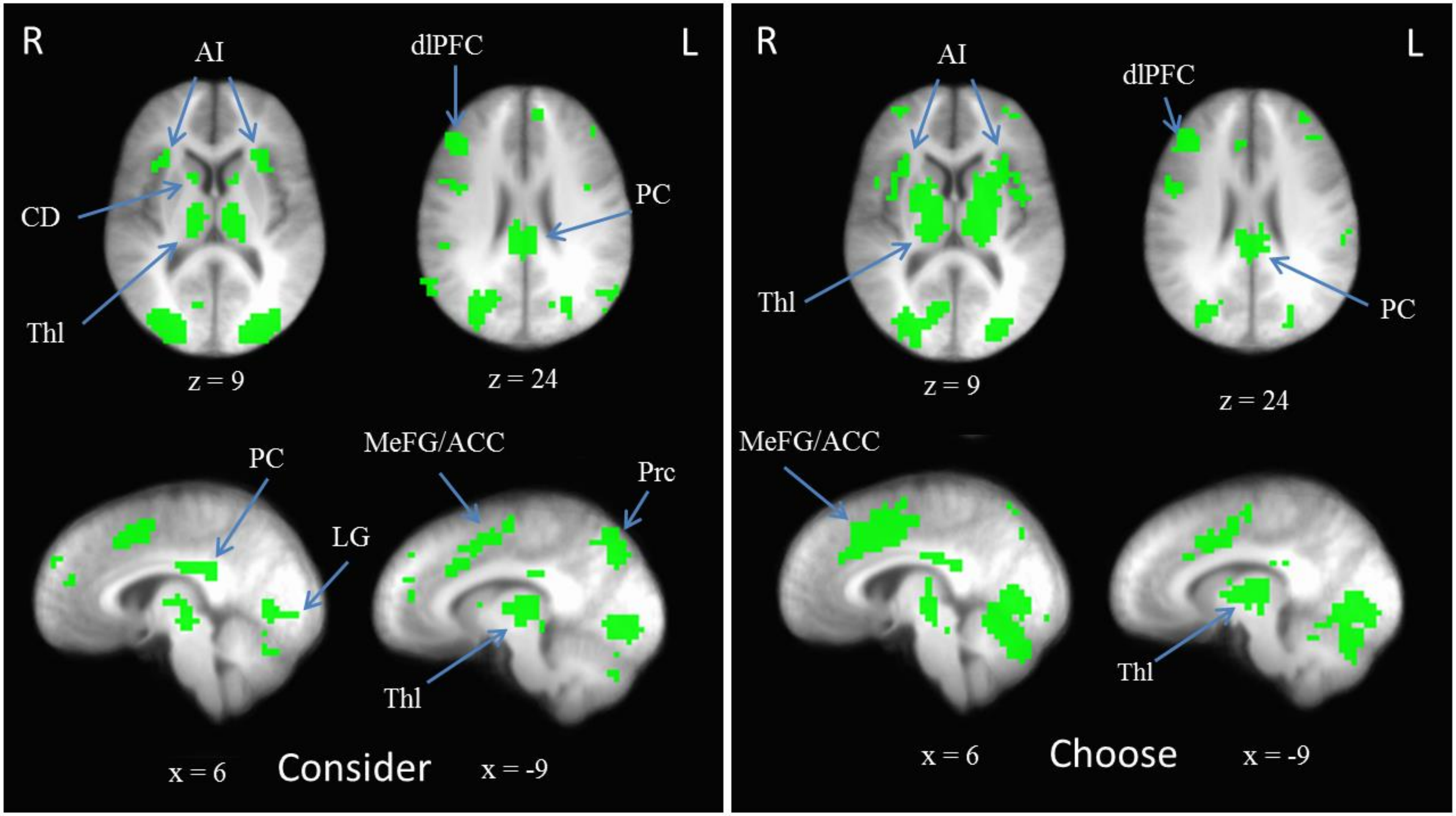

## 2. Supplementary Tables

**Supplementary Table 1. Cigarette Purchase Task fMRI paradigm price sequences.**

| | Price per cigarette | | | |
|---|---|---|---|---|
| **Trial #** | **Run 1** | **Run 2** | **Run 3** | **Run 4** |
| 1 | $0.01 | $0.04 | $0.40 | $0.14 |
| 2 | $0.05 | $0.20 | $2.50 | $0.04 |
| 3 | $0.15 | $0.01 | $0.10 | $0.21 |
| 4 | $0.25 | $0.40 | $0.00 | $0.29 |
| 5 | $0.50 | $0.10 | $0.09 | $0.34 |
| 6 | $2.50 | $0.30 | $0.15 | $1.00 |
| 7 | $0.22 | $0.21 | $0.25 | $0.02 |
| 8 | $0.02 | $0.26 | $0.34 | $0.19 |
| 9 | $2.00 | $0.35 | $10.00 | $0.24 |
| 10 | $0.28 | $2.00 | $0.10 | $0.22 |
| 11 | $5.00 | $0.00 | $0.28 | $0.35 |
| 12 | $0.19 | $2.50 | $0.35 | $0.01 |
| 13 | $0.00 | $0.14 | $0.26 | $0.50 |
| 14 | $5.00 | $0.22 | $0.02 | $2.00 |
| 15 | $0.40 | $0.29 | $0.19 | $0.30 |
| 16 | $0.09 | $0.50 | $0.30 | $10.00 |
| 17 | $0.20 | $1.00 | $0.23 | $0.05 |
| 18 | $0.24 | $0.04 | $0.14 | $0.00 |
| 19 | $0.34 | $0.05 | $0.20 | $0.09 |
| 20 | $0.26 | $0.23 | $0.29 | $0.15 |
| 21 | $1.00 | $0.24 | $0.28 | $0.25 |
| 22 | $0.21 | $10.00 | $5.00 | $0.23 |

**Supplementary Table 2.** ***Consider*** **epoch ROIs revealing differential activation by choice type.**

| **Voxel size (3.5 mm$^3$)** | **X** | **Y** | **Z** | **Region** | ***p*** | **INE vs. ELA *p*** | **INE vs. SUP *p*** | **ELA vs. SUP *p*** |
|---|---|---|---|---|---|---|---|---|
| 14 | -16 | -18.8 | 3.2 | R caudate (‘Smoke’) | 4.42E-6 | .123 | 3.57E-4 | 9.56E-6 |
| 11 | 11.3 | -19.7 | 3.3 | L caudate (‘Smoke’) | 4.76E-6 | .110 | 1.89E-4 | 1.36E-5 |
| 19 | -23.3 | 65.3 | 45.6 | R superior parietal lobule (‘Smoke’) | 3.77E-5 | .055 | 3.08E-6 | .017 |
| 29 | -0.3 | -15.7 | 42.6 | BL medial frontal gyrus (‘Smoke?’) | 4.15E-7 | 2.34E-5 | .153 | 9.62E-5 |
| 6 | 29.8 | 59.3 | -30.8 | L cerebellar tonsil (‘Smoke?’) | 4.76E-6 | 7.24E-6 | .977 | 1.34E-4 |
| 46 | 55.7 | 54.4 | 20.3 | L superior temporal gyrus (‘Smoke?’) | 1.09E-8 | 4.97E-9 | .358 | 2.64E-5 |
| 16 | -27.6 | 1.4 | 51.6 | R middle frontal gyrus (‘Distinct’) | 3.78E-6 | 7.93E-6 | .001 | .047 |
| 35 | 10.1 | 74.5 | -4.4 | L lingual gyrus (‘Restraint’) | 5.24E-7 | 3.37E-5 | 4.34E-6 | .477 |
| 5 | -46.5 | 33.2 | 44.3 | R inferior parietal lobule (‘Restraint’) | 1.72E-5 | 2.80E-4 | 1.75E-5 | .846 |
| 20 | 37.5 | 25.8 | 51.7 | L postcentral gyrus (‘Restraint’) | 1.20E-5 | 3.53E-5 | .001 | .258 |

**Supplementary Table 3.** ***Choose*** **epoch ROIs revealing differential activation by choice type.**

| **Voxel size (3.5 mm$^3$)** | **X** | **Y** | **Z** | **Region** | ***p*** | **INE vs. ELA *p*** | **INE vs. SUP *p*** | **ELA vs. SUP *p*** |
|---|---|---|---|---|---|---|---|---|
| 54 | -44.6 | -33.5 | 21.5 | R middle frontal gyrus (‘Smoke?’) | 6.02E-7 | 1.22E-5 | 1.000 | 4.43E-6 |
| 47 | -28 | -1.5 | 49.8 | R middle frontal gyrus (‘Smoke?’) | 9.86E-7 | 5.24E-5 | .616 | 2.53E-5 |
| 11 | -46.3 | -5 | 21.5 | R inferior frontal gyrus (‘Smoke?’) | 5.14E-6 | 2.56E-4 | .723 | 2.54E-6 |
| 30 | -33.4 | -19.8 | 3.2 | R anterior insula (‘Smoke?’) | 1.11E-5 | 5.92E-5 | .883 | 1.16E-4 |
| 12 | 30.6 | -21.5 | 3.6 | L anterior insula (‘Smoke?’) | 2.36E-4 | .001 | .792 | .002 |
| 91 | -3.2 | -17.8 | 41.1 | BL medial frontal gyrus/ anterior cingulate cortex (‘Smoke?’) | 5.58E-7 | 3.02E-6 | .773 | 1.20E-5 |
| 84 | -42.5 | 42.8 | 37.8 | R inferior parietal lobule (‘Smoke?’) | 2.18E-8 | 5.21E-6 | .838 | 6.19E-8 |
| 11 | 42.2 | 46.8 | 43.7 | L inferior parietal lobule (‘Smoke?’) | 4.81E-5 | .001 | .965 | 9.73E-5 |
| 15 | 50.5 | 28.3 | 22.4 | L inferior parietal lobule (‘Smoke?’) | 1.37E-5 | 2.38E-5 | .763 | 3.29E-4 |
| 15 | -44.5 | 62.6 | 1.1 | R middle temporal gyrus (‘Smoke?’) | 8.04E-6 | 2.61E-5 | .283 | 3.29E-4 |
| 9 | 55.8 | 62.8 | 22.1 | L superior temporal gyrus (‘Smoke?’) | 1.01E-6 | 3.82E-5 | .263 | 3.68E-5 |
| 14 | 6.5 | 54.5 | 20.8 | L posterior cingulate cortex (‘Smoke?’) | 3.91E-5 | .004 | .101 | 1.39E-5 |
| 5 | -1 | -56.4 | -2.6 | BL medial frontal gyrus (‘Smoke?’) | 1.40E-4 | 1.70E-5 | .709 | .002 |
| 13 | 43.8 | -9 | 34.2 | L middle frontal gyrus (‘Distinct’) | 8.91E-6 | .004 | .023 | 2.24E-5 |
| 62 | 34.9 | 28.6 | 54.7 | L postcentral gyrus (‘Distinct’) | 1.01E-6 | 8.11E-7 | .006 | .008 |
| 6 | -45.5 | 15.6 | 53.2 | R postcentral gyrus (‘Distinct’) | 1.59E-5 | 1.50E-4 | .012 | .005 |
| 255 | -13.8 | 63 | -6 | R lingual gyrus (‘Restraint’) | 1.12E-12 | 6.10E-9 | 1.87E-8 | .568 |
| 187 | 12.2 | 70.2 | -6.6 | L lingual gyrus (‘Restraint’) | 1.82E-12 | 5.81E-10 | 2.39E-8 | .164 |
| 47 | -19.5 | 69.6 | 48.2 | R precuneus (‘Restraint’) | 4.22E-7 | 1.40E-6 | 3.07E-4 | .198 |
| 14 | 23.8 | 77.8 | 13.8 | L middle occipital gyrus (‘Restraint’) | 3.17E-5 | 4.82E-4 | 4.95E-4 | .340 |
